# Activation of cGAS-STING Pathway Is Associated with MSI-H Stage IV Colorectal Cancer

**DOI:** 10.3390/cancers15010221

**Published:** 2022-12-30

**Authors:** Nenad Kunac, Marina Degoricija, Jelena Viculin, Jasminka Omerović, Janoš Terzić, Katarina Vilović, Jelena Korac-Prlic

**Affiliations:** 1 Department of Pathology, Forensic Medicine and Cytology, University Hospital Centre Split, 21000 Split, Croatia; 2 Department of Medical Chemistry and Biochemistry, School of Medicine, University of Split, 21000 Split, Croatia; 3 Department of Oncology, University Hospital Centre Split, 21000 Split, Croatia; 4 Laboratory for Cancer Research, Department of Immunology, School of Medicine, University of Split, 21000 Split, Croatia; 5 School of Medicine, University of Split, 21000 Split, Croatia

**Keywords:** colorectal cancer, cGAS-STING pathway, microsatellite instability, immune checkpoint inhibitors, biomarkers

## Abstract

**Simple Summary:**

Metastatic colorectal cancer is one of the most common causes of cancer-related mortality in adults. New therapeutic strategies are currently being implemented for the treatment of colorectal cancer which require new predictive markers. Metastatic colorectal cancer often presents microsatellite instability which is associated with a positive response to immune therapy. The findings of the present study demonstrate and discuss the DNA sensor cGAS and cyclic GMP–AMP receptor stimulator of interferon genes (STING) as potential new targets for novel therapeutic approaches based on immune checkpoint inhibitors in microsatellite unstable stage IV colorectal cancer.

**Abstract:**

Colorectal cancer is the second most common cause of cancer-related mortality in adults. Understanding colorectal tumorigenesis at both the cellular and molecular levels is crucial for developing effective treatment options. Forty-one biopsy samples from patients with metastatic CRC (mCRC) were collected at Split University Hospital in Croatia. A total of 41 patients (21 with microsatellite unstable tumours and 20 with microsatellite stable tumours) were randomly included in the study. Immunolabelling of cGAS and STING in metastatic CRC was performed and further complemented by histological classification, tumour grade, and KRAS, NRAS, and BRAF mutational status of mCRC. In bivariate analysis, elevated expression of cGAS and STING was positively associated with MSI-H colon cancer (Fisher’s exact test, both *p* = 0.0203). Combined expression analysis of cGAS and STING showed a significantly higher percentage of patients with mCRC MSI-H with a fully or partially activated cGAS-STING signalling pathway (chi-square test, *p* = 0.0050). After adjusting for age, sex, and STING expression, increased cGAS expression remained significantly associated with MSI-H colon cancer in a multiple logistic regression model (β = 1.588, SE = ±0.799, *p* = 0.047). The cGAS-STING signalling axis represents a compelling new target for optimization of immune checkpoint inhibitor therapeutic approaches in patients with MSI-H stage IV CRC.

## 1. Introduction

Colorectal cancer ranks second among the leading causes of cancer-related deaths worldwide after lung cancer [1]. According to GLOBOCAN, colorectal cancer was responsible for 9.4 million deaths in 2020, and 1.9 million new cases were estimated in the same year [2]. A considerable number of new cases continues to increase, and this number is predicted to reach 3.2 million in the next 20 years [3]. Although the trends in survival have improved overall, treatment options for metastatic colorectal cancer remain a challenge; approximately 50% of patients eventually develop metastatic disease, and 35% of patients already present with metastatic disease at diagnosis [4,5]. In CRC, the genomic alterations found in primary tumours are likely to be comparable to those in advanced metastatic cells. Sequencing analyses of colorectal cancer suggest metastatic potential in the primary tumour, challenging the general paradigm of metastasis as the evolutionary success of the biological process of individual cells [6].

According to specific molecular and morphological genetic alterations, three major types of molecular alterations occur in CRC, either separately or in different combinations [7]. The most common is chromosomal instability (CIN), which accounts for 65–70% of all sporadic mutations. Loss-of-function mutations in tumour suppressor genes (APC, SMAD4, SMAD4, and TP53) and/or gain of the GTP-ase activity of Ras protein play a key role in driving CRC development. The second mechanism, which contributes to the altered epigenome in CRC, is attributed to CpG island methylation of tumour suppressor gene promotors and histone modifications. Promoter hypermethylation of mismatch repair genes MLH1, MSH2, MSH6, and PMS2 reduces the level of expression of these genes, causing another molecular alteration in CRC, known as microsatellite instability (MSI) [8]. Other malignancies, including hereditary colon, gastric, and sporadic endometrial cancers, are also characterized by MSI. They are present in approximately 15–25% of stage II and III and 3–4% of stage IV colorectal malignancies. The prognostic and therapeutic consequences of MSI status identification have been used for diagnostic tumour detection and categorization. These findings emphasize the importance of microsatellite instability in CRC patients [9].

Therapeutic strategies for CRC involve screening for several genetic abnormalities that are prognostic or predictive biomarkers of CRC. Response to anti-epithelial growth factor receptor therapy (anti-EGFR) is predicted by RAS gene family members, including KRAS and NRAS, and it has been proposed that BRAF mutations also affect the prognosis of mCRC [10]. Activating mutations in the RAS and RAF genes lead to aberrant signalling downstream of the EGFR receptor, including persistent MAPK stimulation, resulting in uncontrolled cell proliferation, leading to cancer [11]. KRAS mutations are found in 42% of CRC cases, whereas NRAS and BRAF mutations are less common [12]. Furthermore, mutations in KRAS, NRAS, and BRAF are mutually exclusive in colorectal tumours, and it has been reported that patients with BRAF and NRAS modifications have considerably shorter survival rates.

In addition to this, the expression of the aforementioned biomarkers, in conjunction with the MSI status of the tumour serves as a predictor of successful immunotherapy with anti-EGFR or immune checkpoint inhibitors (ICIs). Patients with mCRC presenting with MSI-H or deficient mismatch repair (dMMR) are currently the only known good responders to immunotherapy with ICIs [13], which has been approved as a first-line therapy for this group of patients. Although MSI status is a good predictive marker, immunotherapy in patients with MSI-H mCRC has an overall response rate of only 44%. Therefore, depicting specific additional predictive markers and/or gene expression signatures will benefit rational treatment strategy for mCRC [14].

The cGAS-STING pathway is an innate immune cytosolic double-stranded DNA (dsDNA) sensor that is important for the response to pathogen infection and inflammation. Moreover, the cGAS-STING pathway is responsible for the innate immune detection of cancer, thus playing an important role in anti-cancer immunity, as well as potentiating the effects of cancer immunotherapy [15]. Tumours with mutated MMR genes are known to induce cGAS-STING signalling. Loss of the MutLα subunit of MLH1 generates chromosomal abnormalities and the release of nuclear DNA into the cytoplasm, thus activating cGAS-STING signalling [16]. In addition, Kaneta et al. recently demonstrated that the downregulation of genes responsible for DNA mismatch repair enhances the activation of the cGAS-STING pathway, which is important for the recruitment of CD8+ cells in the CRC tumour microenvironment [17]. Therefore, the goal of our study was to assess the expression levels of cGAS and STING in mCRC, both within microsatellite stable and unstable groups of patients with stage IV CRC. These findings were further complemented by the histological classification, tumour grade, and KRAS, NRAS, and BRAF mutational status of mCRC.

## 2. Methods

### 2.1. Patient Samples

Forty-one biopsy samples from metastatic CRC patients were collected at the Department of Pathology, Split University Hospital, Split, Croatia from January 2020 to December 2021. Clinicopathological data were collected from the Department of Oncology, Split University Hospital. Twenty-one patients with microsatellite unstable tumours and twenty patients with microsatellite stable tumours were randomly included in the study. Histological classification of tumour grade was performed using hematoxylin and eosin–stained sections. Tumours were staged as low- and high-grade tumours, as recommended by a multidisciplinary colorectal working group of a Consensus Conference, sponsored by the College of American Pathologists. According to this system, stratification was based solely on the proportion of gland formation by the tumour: low-grade with <50% gland formation and high-grade with ≥50% gland formation.

The study was approved by the Ethics Committees of the Clinical Hospital Center and Medical School Split (Klasa: 500-03/19-01/36, Ur.br.: 2181-147-01/06/M.S.+19-2). All patients provided signed informed consent for all therapeutic and diagnostic procedures. All methods were performed according to the relevant guidelines and regulations.

### 2.2. Molecular Analysis

Mutational status of metastatic colorectal cancer was determined for KRAS, NRAS, and BRAF by RT PCR Cobas Z480 (Roche, Basel, Switzerland), using Cobas KRAS Mutation Test v2 and BRAF/NRAS FFPET Mutation Test. Microsatellite instability of the tumour tissue was determined using the Idylla MSI test (Biocartis, Jersey City, NJ, USA).

### 2.3. Immunohistochemistry

Immunohistochemical staining was performed on formalin-fixed, paraffin-embedded representative tissue sections of 5 µm thickness. The slides were dried overnight at 60 °C and deparaffinized in xylene and rehydrated using graded alcohol solutions in water. Heat-induced epitope retrieval was performed by boiling the sections in EDTA buffer (pH 8.9) in a microwave oven at 1000 W for 20 min (4 times per 5 min each). After boiling, the sections were left to cool at room temperature for 20 min, rinsed thoroughly with water, and placed in Tris-buffered saline (TBS) for 5 min. Endogenous peroxidase was blocked with a peroxidase block solution (EnVision kit, Dako-Cytomation, Glostrup, Denmark) for 15 min, and the slides were rinsed with TBS. The sections were incubated for 1 h and 30 min with either primary mouse polyclonal anti-human c-GAS antibody (Proteintech, 26416-1-AP, dilution 1:200), STING antibody (Proteintech, 19851-1-AP, dilution 1:2000), CD4 antibody (SP35, 790-4423, Ventana), or CD8 antibody (SP57, 790-4460, Ventana). After rinsing in TBS buffer, the slides were incubated with secondary antibodies and visualized using the OptiView DAB IHC v6 procedure. The slides were counterstained with hematoxylin, dehydrated, and mounted.

### 2.4. Immunohistochemical Staining

**Figure 1:**
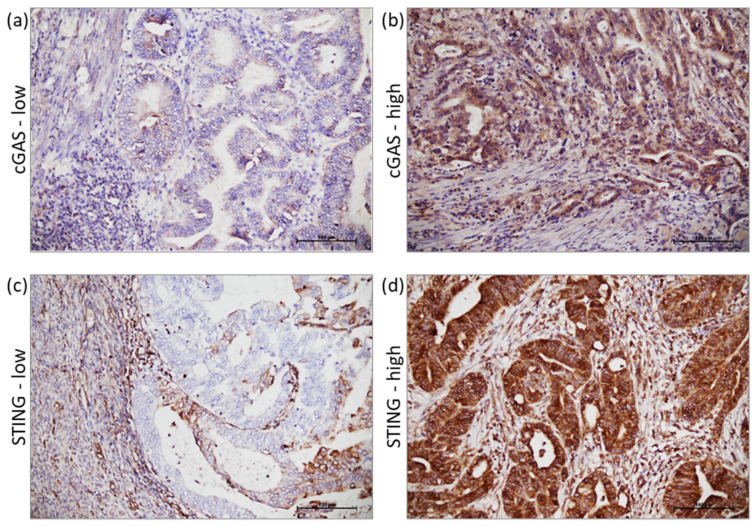
cGAS and STING immunostaining. Representative histologic images of low and high expression of cGAS (**a**) and (**b**) and STING (**c**) and (**d**), respectively.

Two independent pathologists semi-quantitatively assessed the IHC expression levels of cGAS and STING proteins by integrating the percentage and intensity of immunostaining of the cancer cells. Immunoreactivity intensity was marked from 0 to 3+. H-score was generated by using the formula ΣP*i*(*i* +1), where *i* is intensity of immunostaining and P*i* is percentage of cancer cells. Cut-offs for high and low expression levels were defined for each protein. For cGAS staining, high expression was defined for samples having a minimum of 20% cancer cells with an intensity of immunostaining 2+ and/or 3+, and for STING staining, high expression was defined for samples with more than 50% cancer cells with an intensity of immunostaining 2+ and/or 3+ (Figure 1).

### 2.5. Statistics

Continuous data were presented as medians with interquartile ranges, whereas categorical variables were presented as whole numbers and percentages. The independence of categorical variables was tested using Fisher’s exact test and chi-square (χ^2^) test. Spearman’s correlation coefficient was calculated to test the associations between categorical variables. In addition to this, multiple logistic regression was used to model the probability of increased STING and cGAS expression with microsatellite instability in colon cancer. Statistical significance was defined as a two-tailed *p* < 0.05. Data were analysed using GraphPad Prism (version 9.4.0., La Jolla, CA, USA).

## 3. Results

A total of 41 biopsy samples from stage IV CRC patients were collected: 21 patients with microsatellite unstable tumours (MSI-H) and 20 patients with microsatellite stable tumours (MSS) were randomly included in the study. The median age of mCRC patients was 66 years (range, 34 to 83) with a higher proportion of patients aged ≥60 years. The male and female proportions of enrolled patients were 59% and 41%, respectively. The detailed clinicopathological characteristics of the stage IV CRCs patients included in the study are presented in Table 1 and Table S1 and are in accordance with previously published patient cohorts for stage IV CRC [18].

The location of cancer in the ascending (RC) colon was positively associated with the MSI-H type of colon cancer (chi-square test, *p* ≤ 0.0001). In addition, a mild positive correlation was observed between high-grade tumours and the ascending (right colon, RC) colon (Spearman R = 0.55, *p* = 0.0002). Furthermore, the present study also determined a substantial mutational load of KRAS, NRAS, and BRAF in patients with both MSI-H and MSS.

In bivariate analysis, elevated expression of cGAS and STING in cancer cells was positively associated with MSI-H colon cancer (Fisher’s exact test, both *p* = 0.0203).

**Figure 2:**
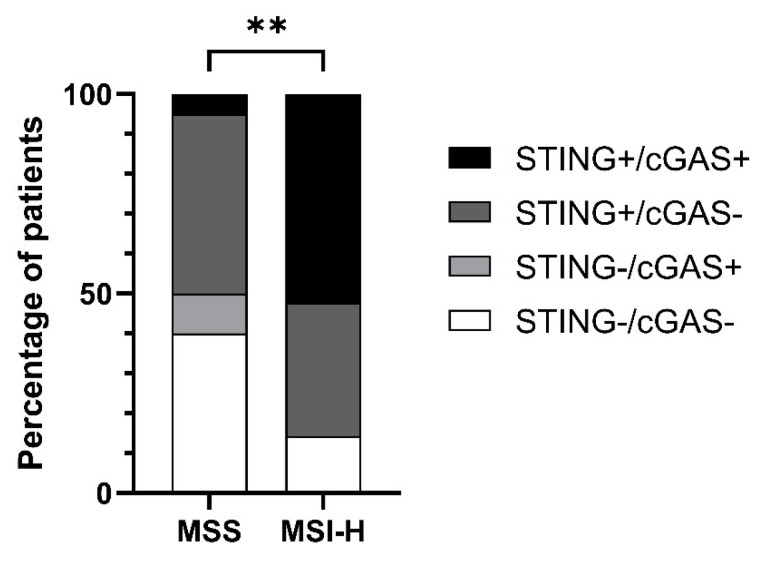
IHC analysis of STING and cGAS expression status in MSS and MSI-H. Chi-square test, ** *p* = 0.0050.

Combined expression analysis of cGAS and STING showed a significantly higher percentage of patients with MSI-H mCRC with a fully or partially activated cGAS-STING signalling pathway in cancer cells (chi-square test, *p* = 0.0050) (Figure 2). Analysis of cGAS and STING H-scores showed a mild positive correlation between cGAS and STING expression (Spearman R = 0.38, *p* = 0.0138). Furthermore, the immune cell composition was always represented as a heterogeneous population of positive and negative staining cells for cGAS and STING.

Individual mutation rates of KRAS, NRAS, and BRAF were not associated with STING’s expression status. However, the frequency of all three mutations was significantly higher in tumours with low STING expression (Fisher’s exact test, *p* = 0.0425) (Table 2).

After adjusting for age, sex, and STING expression, increased cGAS expression remained significantly associated with MSI-H colon cancer in the multiple logistic regression model (β = 1.588, SE = ±0.799, *p* = 0.047).

**Figure 3:**
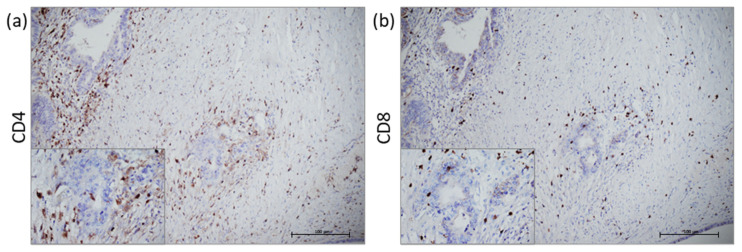
CD4 and CD8 immunostaining. Representative histologic images of CD4^+^ (**a**) and CD8^+^ immune cells (**b**).

MSI-H and MSS tumours were additionally analysed for T cell infiltration. All the samples demonstrated positive staining for CD4^+^ and CD8^+^ cell infiltrates diffused in tumour stroma (Figure 3).

## 4. Discussion

Understanding tumorigenesis at both cellular and molecular levels is crucial for developing effective treatment options for CRC characterized by a deficient mismatch repair system (dMMR) which causes microsatellite instability (MSI). Tailored therapeutic approaches have become easier in CRC owing to a growing understanding of the molecular pathways underlying this malignancy. The objective of the present study was to determine the expression of the DNA sensor cGAS and cyclic GMP–AMP receptor stimulator of interferon genes (STING) in stage IV CRC with respect to the presence of microsatellite instability (MSI-H). One of the most extensively researched nucleic acid-sensing pattern recognition receptors (PRRs), STING is essential for regulating antiviral activities and detecting tumour development [19]. The cytoplasmic nucleotide transferase cGAS senses tumour-derived DNA in the cytoplasm of dendritic cells and further catalyses the synthesis of cyclic GMP–AMP (GAMP) to bind and trigger STING, which then promotes type I IFN responses to initiate antitumour responses and enhance CD8^+^ T cell cross-priming [20]. Recent research has revealed that STING plays a role in carcinogenesis and treatment resistance, proposing STING as a potentially effective therapeutic target in patients with CRC [21]. An improved prognosis for CRC patients is associated with the activation of the STING signalling system [22,23].

Consequently, a compromised STING pathway may impede T cell priming and fail to identify tumour-associated antigens. Enhancing or activating the STING pathway creates an opportunity for improving the anti-tumour immunotherapy. In the present study, elevated expression of cGAS and STING was positively associated with MSI-H colon cancer. A significant loss of cGAS and STING expression has been reported in later stages of colon cancer; moreover, it has been demonstrated that cGAS expression is lost in the earlier stages in comparison to STING expression [23,24]. Our results also demonstrate a significant fraction of tumours that display STING expression in the absence of cGAS staining. Even though STING expression is generally lost in stage IV CRC, our data demonstrate that expression of STING is preserved in patients with MSI-H CRC. In line with our findings, previous analysis of the TCGA database and subsequent IHC analysis showed elevated expression of cGAS and STING in MSI-H Stage I–IV CRC [17].

Higher cGAS expression appears to be a prognostic factor, associated with prolonged disease-free survival and overall survival in MSI-H tumours [25]. In addition to this, elevated STING expression is associated with a good response to immunotherapy [26]. These data suggest that the cGAS-STING pathway is an important pathway for enhancing the response to ICIs in patients with MSI-H tumours [27]. Since elevated cGAS and STING expression is considered a marker for a good immunotherapy response, our data also suggest that patients with MSI-H mCRC represent potential responders for the ICI therapeutic approach.

In line with previous findings corroborating that patients with MSI-H colon cancer present with distinct clinical features, the dominant being the proximal location with poor differentiation, our study also demonstrated a positive association between MSI-H cancer and right-sided localization [9,28]. In addition, a mild positive correlation was also observed between high-grade tumours and ascending (RC) colon cancers. Our findings are in support of previous studies, indicating that patients with tumours localized in the right colon frequently have larger, more advanced, and poorly differentiated tumours. The genetic background of right CRC and left CRC is diverse. Patients with right CRC typically have tumours with a higher rate of microsatellite instability (MSI), whereas patients with left CRC have tumours with a higher rate of chromosomal instability [29].

It has been demonstrated that the expression of biomarkers such as KRAS, NRAS, and BRAF, in conjunction with the MSI state of the tumour, serves as a negative predictor for anti-EGFR therapy in mCRC patients [11,26]. The present study also demonstrated the substantial presence of KRAS, NRAS, and BRAF-activating mutations in both MSI-H and MSS patient populations. The function of the RAS family of proteins, which are members of the GTPase protein group present in all cellular organisms, is to convey signals between the cells which promote cell division. KRAS and NRAS proteins are two primary members of the RAS family. The clinical and pathological characteristics of our patients with KRAS and NRAS mutations were identical to those reported in the literature [30].

Although KRAS, NRAS, and BRAF mutations are negative predictive markers for anti-EGFR therapy, recent studies have shown KRAS and NRAS mutational status is not predictive for a successful ICI therapeutic approach [14,31]. Furthermore, in KRAS mutant cancers, immune checkpoint molecules are downregulated [32]. On the contrary, STING induces PD-L1 expression [33]. Consistent with these findings, the frequency of these mutations in our patients was significantly higher in tumours with low STING expression levels. Suppression of STING in KRAS-driven lung cancer is associated with LKB1 loss, thus, it would be interesting to analyse whether mCRC with KRAS mutations also has a mutation in the LKB1 gene [34].

The STING pathway can also be activated by STING agonists, which can change the tumour microenvironment by inducing IFN-β production, which is necessary for productive CD8^+^ T cell cross-priming against tumour-associated antigens [19]. Kaneta et al. demonstrated that stimulation of CRC cells with a STING agonist results in elevated migration potential of CD8^+^ lymphocytes from peripheral blood through production of CCL5 and CXCL10 chemokines [17]. Our data support previous findings, demonstrating both CD8^+^ and CD4^+^ positive T cells in tumour stroma of stage IV CRC.

Since STING increases PD-L1 expression, preclinical and phase I trial data have demonstrated the potential of combined STING agonist and immunotherapy. To date, there has also been a phase II clinical trial for head and neck squamous cell carcinoma with combination therapy including STING agonist and pembrolizumab (NCT04220866) [15]. Patients with MSI-H stage IV CRC could also benefit from this therapeutic approach, as could the patients with MSS stage IV CRC who are non-responders to pembrolizumab [14].

## 5. Conclusions

Mutations in DNA mismatch repair genes observed in sporadic colorectal malignancies cause microsatellite instability (MSI) and the MSI-H phenotype which predicts better overall survival. Biomarker monitoring in patients with mCRC has become a standard procedure in the clinical setting. Since new therapeutic strategies are currently being implemented for the treatment of mCRC, new predictive markers are required. cGAS and STING expression in tumour cells might have a predictive potential for successful immunotherapy in patients with MSI-H stage IV CRC and future prospective studies should evaluate these findings. In addition to this, STING-targeted cancer immunotherapy could alter the tumour immune microenvironment of mCRC to improve anticancer immunity. Our findings may aid the future customization of the therapeutic approach for stage IV CRC, enhancing overall survival and considerably reducing drug-related toxicities.

## Figures and Tables

**Table 1:** Clinicopathological characteristics of patients with stage IV CRC.

Characteristic	Overall, N = 41	MSI-H, N = 21	MSS, N = 20	*p*-Value
**Age**, years (IQR)	66.0 (56.0–74.5)	66.0 (50.5–74.5)	66.5 (58.3–75.5)	
				0.7442 ^§^
<60	14 (34%)	8 (38%)	6 (30%)	
≥60	27 (66%)	13 (62%)	14 (70%)	
**Gender**				0.7557 ^‡^
Male	24 (59%)	13 (62%)	11 (55%)	
Female	17 (41%)	8 (38%)	9 (45%)	
**Side**				**<0.0001 ^§^**
RC	21 (51%)	18 (85%)	3 (15%)	
LC	12 (29%)	1 (5%)	11 (55%)	
R	8 (20%)	2 (10%)	6 (30%)	
**Grade**				0.1809 ^§^
Low	28 (68%)	12 (57%)	16 (80%)	
High	13 (32%)	9 (43%)	4 (20%)	
**KRAS**				>0.9999 ^§^
Wild type	33 (80%)	17 (81%)	16 (80%)	
Mutant	8 (20%)	4 (19%)	4 (20%)	
**NRAS**				0.3433 ^§^
Wild type	37 (90%)	20 (95%)	17 (85%)	
Mutant	4 (10%)	1 (5%)	3 (15%)	
**BRAF**				0.1836 ^§^
Wild type	35 (85%)	16 (76%)	19 (95%)	
Mutant	6 (15%)	5 (24%)	1 (5%)	
**cGAS**				**0.0203 ^§^**
Low	27 (66%)	10 (48%)	17 (85%)	
High	14 (34%)	11 (52%)	3 (15%)	
**STING**				**0.0203 ^§^**
Low	13 (32%)	3 (15%)	10 (50%)	
High	28 (68%)	18 (85%)	10 (50%)	

MSI-H: High-frequency microsatellite instability; MSS: microsatellite stable; RC: right colon; LC: left colon; R: rectum; KRAS: V-Ki-Ras2 Kirsten rat sarcoma 2 viral oncogene homolog; NRAS: neuroblastoma RAS viral oncogene homology; BRAF: v-Raf murine sarcoma viral oncogene homolog B; cGAS: cyclic GMP-AMP synthase; STING: stimulator of IFN genes. ^§^ Fisher’s exact test, ^‡^ Chi-square test. The *p*-value represented in bold is statistically significant.

**Table 2:** Rates for mutation of KRAS, NRAS, and BRAF by STING expression status.

Characteristic	Overall, N = 41	STING		*p* Value
		High N = 28	Low N = 13	
**KRAS**				0.0840 ^§^
Wild type	33 (81%)	25 (89%)	8 (62%)	
Mutant	8 (19%)	3 (11%)	5 (38%)	
**NRAS**				0.5795 ^§^
Wild type	37 (90%)	26 (93%)	11 (85%)	
Mutant	4 (10%)	2 (7%)	2 (15%)	
**BRAF**				>0.9999 ^§^
Wild type	35 (85%)	24 (86%)	11 (85%)	
Mutant	6 (15%)	4 (14%)	2 (15%)	
**KRAS + NRAS + BRAF**				**0.0425 ^§^**
Wild type	23 (56%)	19 (68%)	4 (31%)	
Mutant	18 (44%)	9 (32%)	9 (69%)	

KRAS: V-Ki-Ras2 Kirsten rat sarcoma 2 viral oncogene homolog; NRAS: neuroblastoma RAS viral oncogene homolog; BRAF: v-Raf murine sarcoma viral oncogene homolog B; STING: stimulator of IFN genes. ^§^ Fisher’s exact test. The *p*-value represented in bold is statistically significant.

## Data Availability

The data presented in this study are available in this article and Supplementary Material.

## Supplementary Materials

The following supporting information can be downloaded at: https://www.mdpi.com/article/10.3390/cancers15010221/s1, Table S1: Detailed clinicopathological characteristics of patients with stage IV CRC.
