# Supplementary material for: Activation of cGAS-STING Pathway Is Associated with MSI-H Stage IV Colorectal Cancer
Source: Cancers (Basel). 2022 Dec 30;15(1):221. doi: 10.3390/cancers15010221 (PMC9818394; doi:10.3390/cancers15010221)
Supplement: Supplementary file 1 [file cancers-15-00221-s001.zip › cancers-2104534-supplementary.pdf]

Table S1. Detailed clinicopathological characteristics of patients with stage IV CRC.

Patient No	Gender	Age/years	MSI	Side	Grade	KRAS Mutation	NRAS Mutation	BRAF Mutation	cGAS	STING
1	M	43	H	RC	L	No	No	No	L	H
2	F	75	H	RC	H	No	No	No	H	H
3	M	74	H	RC	H	Yes	No	No	L	L
4	M	52	H	RC	H	No	No	Yes	L	L
5	M	49	H	RC	L	No	No	No	H	H
6	M	83	H	RC	L	No	Yes	No	H	H
7	M	78	H	LC	L	No	No	No	H	H
8	F	66	H	RC	L	Yes	No	No	H	H
9	M	57	H	RC	L	No	No	No	L	H
10	F	65	H	RC	L	No	No	No	H	H
11	F	48	H	RC	H	No	No	No	L	H
12	M	62	H	RC	L	Yes	No	No	H	H
13	F	73	H	RC	H	Yes	No	No	L	L
14	M	57	H	RC	H	No	No	No	H	H
15	M	81	H	RC	L	No	No	Yes	H	H
16	F	34	H	RC	H	No	No	Yes	H	H
17	F	39	H	RC	H	No	No	No	H	H
18	M	67	H	R	L	No	No	Yes	L	H
19	M	69	H	R	L	No	No	No	L	H
20	F	73	H	RC	L	No	No	Yes	L	H
21	M	83	H	RC	H	No	No	No	L	H
22	F	54	S	R	L	No	No	No	L	H
23	F	51	S	R	L	No	No	No	H	L
24	F	74	S	RC	H	No	No	Yes	L	L
25	M	65	S	RC	H	No	Yes	No	H	L
26	M	58	S	LC	L	No	No	No	L	H
27	F	72	S	R	L	Yes	No	No	L	L
28	M	80	S	LC	L	Yes	No	No	H	H
29	M	77	S	RC	H	No	Yes	No	L	H
30	M	63	S	LC	L	No	No	No	L	H
31	M	71	S	LC	L	No	No	No	L	H
32	M	55	S	LC	L	No	No	No	L	H
33	M	43	S	R	L	No	No	No	L	H
34	M	82	S	LC	L	Yes	No	No	L	L
35	M	76	S	LC	L	No	No	No	L	L
36	F	82	S	LC	L	No	No	No	L	L
37	F	68	S	LC	L	No	Yes	No	L	L
38	F	62	S	R	L	No	No	No	L	H
39	M	59	S	LC	H	Yes	No	No	L	L
40	F	70	S	R	L	No	No	No	L	H
41	F	64	S	LC	L	No	No	No	L	L

MSI: H—High-frequency microsatellite instability; S—microsatellite stable; RC—right colon; LC—left colon; R—rectum; KRAS—V-Ki-Ras2 Kirsten rat sarcoma 2 viral oncogene homolog; NRAS—neuroblastoma RAS viral oncogene homology; BRAF—v-Raf murine sarcoma viral oncogene homolog B; cGAS—cyclic GMP-AMP synthase; STING—stimulator of IFN genes; L—low, H—high.
